# Initiation of a Wide QRS Complex Tachycardia with Alternating Ventriculoatrial Interval: What Is the Mechanism?

**DOI:** 10.19102/icrm.2025.17021

**Published:** 2026-02-15

**Authors:** Can Ozkan, Ozcan Ozeke, Elif Hande Ozcan Cetin, Meryem Kara, Sona Huseynova, Ahmet Korkmaz, Firat Ozcan, Serkan Cay, Dursun Aras, Serkan Topaloglu

**Affiliations:** 1 Department of Cardiology, University of Health Sciences, Bursa City Hospital, Bursa, Turkey; 2 Department of Cardiology, University of Health Sciences, Ankara Bilkent City Hospital, Ankara, Turkey; 3 Department of Cardiology, Istanbul Medipol University, Istanbul, Turkey

**Keywords:** Atriofascicular pathway, left Mahaim, wide complex tachycardia

## Abstract

The differential diagnosis for wide complex tachycardia includes all causes of narrow complex tachycardia with bundle branch block, all causes of narrow complex tachycardia with antegrade pre-excitation, ventricular tachycardia, and antidromic and other pre-excited reciprocating tachycardias. The variation in a specific intracardiac interval that causes a subsequent change in the tachycardia cycle length or another intracardiac interval can be diagnostic in these arrhythmias.

## Case presentation

**Figure 1:**
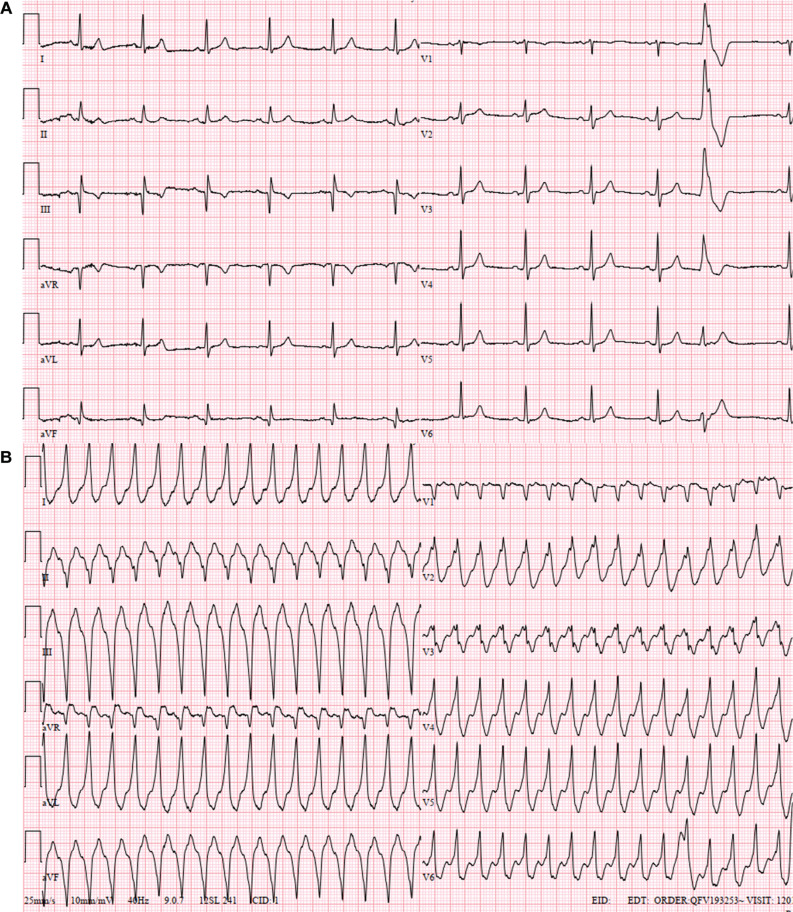
The 12-lead electrocardiograms during sinus rhythm **(A)** and tachycardia **(B)**.

**Figure 2:**
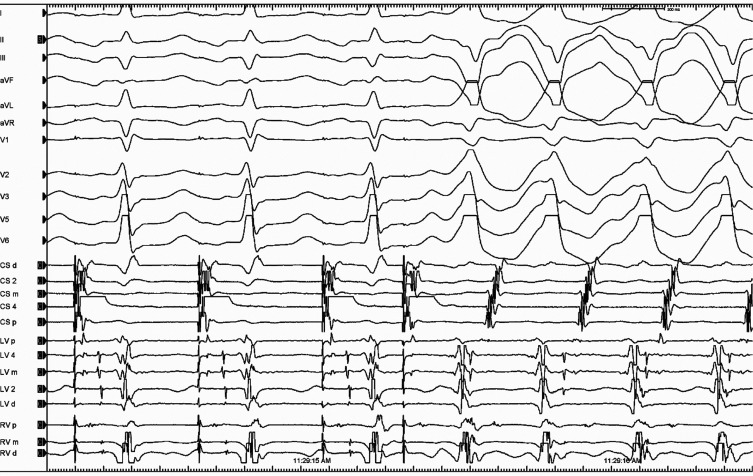
Initiation of the wide complex tachycardia by a programmed atrial stimulation.

A 70-year-old man with recurrent palpitations and no structural heart disease was referred for an electrophysiological study. The patient’s baseline electrocardiogram (ECG) did not show any evidence of pre-excitation **(Figure 1A)**; however, his ECGs taken during tachycardia showed wide QRS complex tachycardia (WCT) with a pattern break appearance in V2 **(Figure 1B)**.^1^ The programmed atrial stimulation initiated the clinical WCT **(Figure 2, Video 1)**. We questioned what the diagnosis might be.

## Discussion

**Figure 3:**
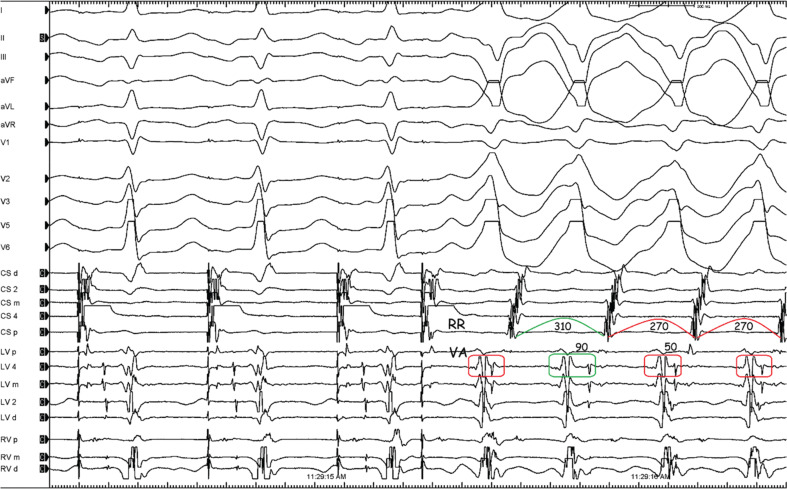
R–R and ventriculoatrial intervals labeled on the tracing shown in **Figure 2**.

In terms of the mechanism for the WCT, the main question that needs to be answered is whether this is ventricular tachycardia (VT), pre-excited tachycardia, or supraventricular tachycardia (SVT) with aberrancy.^1–15^ The first step to understanding tachycardia mechanisms begins with careful assessments of the H–V interval and His sequence during tachycardia.^16^ The lack of a His deflection preceding each QRS complex suggests that activation does not use the infranodal conduction system, making aberrant conduction less likely **(Video 2)**. The WCT with a right bundle branch block (RBBB) morphology and precordial pattern break showed a 1:1 A–V relation and a negative H–V interval, which excludes SVT with RBBB aberrancy **(Figure 2)**,^17^ leaving only two possibilities for the mechanism: pre-excited tachycardia (with active or passive bystander activation) or VT. If retrograde activation of the His bundle has been determined and changes in the V–H or H–A intervals predict changes in the atrial cycle length and reset the tachycardia **(Figures 2 and 3)**, then antidromic tachycardia, either with an atrioventricular bypass tract or an atriofascicular (AF) tract, is present.^18,19^ Therefore, the most striking finding in the present tracing was that there were oscillations in the cycle length and ventriculoatrial intervals **(Figure 3)**, which predicted subsequent changes in A–A intervals implicating the retrograde conduction system in the tachycardia circuit.^6,18^ Therefore, the only option would be the presence of the accessory pathway (AP).

**Figure 4:**
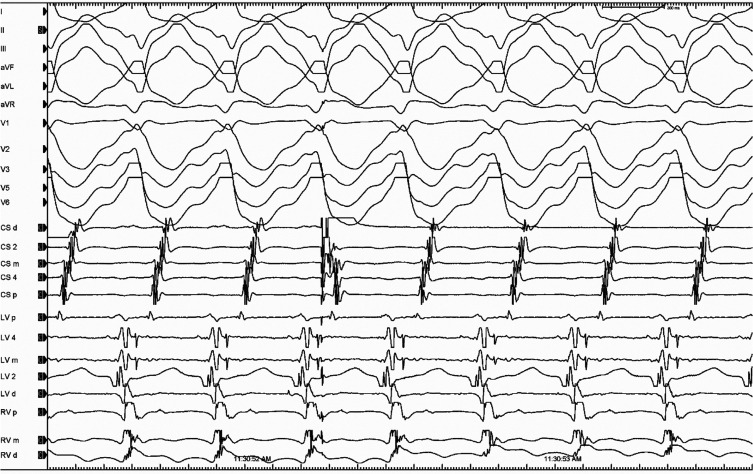
A premature atrial contraction applied from the distal coronary sinus advances the ventricular electrogram without changing the QRS morphology and without advancing the atrial electrogram on the proximal coronary sinus. The advanced ventricular electrogram in turn advances the retrograde His and atrial electrogram without changing the atrial activation sequence.

At first glance, ablation was applied at the site of the earliest ventricular activation; however, it was unsuccessful. Upon closer inspection, a left-sided Mahaim potential was identified (**Video 2**, 20A number 1–2).^20^ A premature atrial contraction applied from the distal coronary sinus advanced the ventricular electrogram without changing the QRS morphology and without advancing the atrial electrogram on the proximal coronary sinus. The advanced ventricular electrogram in turn advanced the retrograde His and atrial electrogram without changing the atrial activation sequence **(Figure 4)**. An A–V interval of ≥150 ms during pre-excited tachycardia is also a fast and reliable method for detecting a decremental conducting AP.^21^ Moreover, the A–V/A–A interval index during tachycardia was >0.55, suggesting a pre-excited tachycardia using a decrementally conducting bypass tract **(Figure 4)**.^21^ The left-sided AF pathway was mapped to the 6 o’clock position of the mitral annulus and successfully ablated **(Video 3)**.

## Supporting information

Supplementary Video 1: Initiation of tachycardia by programmed atrial stimulation. A multipolar catheter was positioned in the proximal His-Purkinje region. 20A 1-12 to 19-20 multipolar Pentaray catheter; REF 1-2 to 9-10 left sided His catheter; CS 1-2 to 9-10 coronary sinus catheter

Supplementary Video 2: Note the M potential (White electrograms, 1-2 numbers) before ventricular activation with retrograde left-sided (White electrograms, 3-4 and 7-8 numbers) and right-sided (yellow electrograms, 3-4 and 5-6 numbers) . 20A 1-12 to 19-20 multipolar Pentaray catheter; REF 1-2 to 9-10 right sided His catheter; CS 1-2 to 9-10 coronary sinus catheter

Supplementary Video 3: Termination of tachycardia at the posterior mitral annulus area
